# Chemo-immunotherapy for metastatic non-squamous NSCLC in a patient with HIV infection: A case report

**DOI:** 10.3389/fonc.2023.1053497

**Published:** 2023-02-02

**Authors:** Alessandro Inno, Emanuela Lattuada, Giovanni Foti, Stefania Gori

**Affiliations:** ^1^ Unità Operativa di Oncologia Medica, IRCCS Ospedale Sacro Cuore Don Calabria, Negrar di Valpolicella (VR), Italy; ^2^ Unità Operativa di Malattie Infettive e Tropicali, AOUI Verona, Verona, Italy; ^3^ Unità Operativa di Radiologia, IRCCS Ospedale Sacro Cuore Don Calabria, Negrar di Valpolicella (VR), Italy

**Keywords:** NSCLC, HIV, chemotherapy, immunotherapy, carboplatin, pemetrexed, pembrolizumab, anti-PD1

## Abstract

Activity and safety data of chemo-immunotherapy for patients with metastatic NSCLC and known HIV infection are still limited, since HIV-positive patients were generally excluded from clinical trials. Here we report the case of a metastatic NSCLC patient with HIV infection and undetectable viral load treated with first-line chemo-immunotherapy (pembrolizumab, carboplatin and pemetrexed), achieving a meaningful and durable objective response, with no treatment-related adverse events and no HIV-related complications. This report suggests that NSCLC patients with virologically controlled HIV infection can be safely treated with chemo-immunotherapy and should not be excluded from this treatment based on their viral infection only.

## Introduction

Immune checkpoint inhibitors (ICIs) have deeply changed the treatment landscape of non-small cell lung cancer (NSCLC) over the last decade. For metastatic, non-oncogene addicted NSCLC, monoclonal antibodies against programmed cell death protein 1 (PD-1) or programmed cell death ligand 1 (PD-L1) now represent standard of care as single-agents in the first-line treatment for patients with PD-L1 expression on ≥50% of tumor cells, or in combination with standard platinum-based chemotherapy for all patients regardless of PD-L1 expression level (1–3). However, clinical trials with ICIs, alone or in combination with chemotherapy for NSCLC, generally excluded patients living with human immunodeficiency virus (HIV) infection (PLWHI), due to the concern of viral flares and/or triggering of immune-related adverse events, therefore the benefit/risk balance of ICIs needs to be further elucidated in this special population (4).

Here we report the case of a patient with HIV infection and metastatic NSCLC treated with pembrolizumab plus chemotherapy.

## Case presentation

In September, 2020 a 50-year-old current smoker male presenting with persisting cough, fatigue and dyspnea was diagnosed with non-squamous, *EGFR* wild-type, ALK negative, ROS1 negative, PDL-1 positive (tumor proportion score 5%) NSCLC. Baseline tumor assessment with head-chest-abdomen CT scan and total-body 18-FDG PET/CT scan showed a large primary tumor (10 cm in size) in the upper right pulmonary lobe with metastatic homolateral hilar, mediastinal, supraclavicular and IIa/Ib level latero-cervical lymph nodes (stage cT4 cN3 cM1a, cIVA). Relevant past medical history included: squamous cell carcinoma of the nasal wing treated with radical surgery followed by adjuvant radiotherapy with concomitant weekly cisplatin chemotherapy in 2016; recurrent breast abscesses; known HIV infection since 2008, on treatment with ART consisting of emtricitabin/rilpivirine/tenofovir at the time of NSCLC diagnosis. In addition, baseline infectious disease screening also revealed past HBV infection.

**Figure 1:**
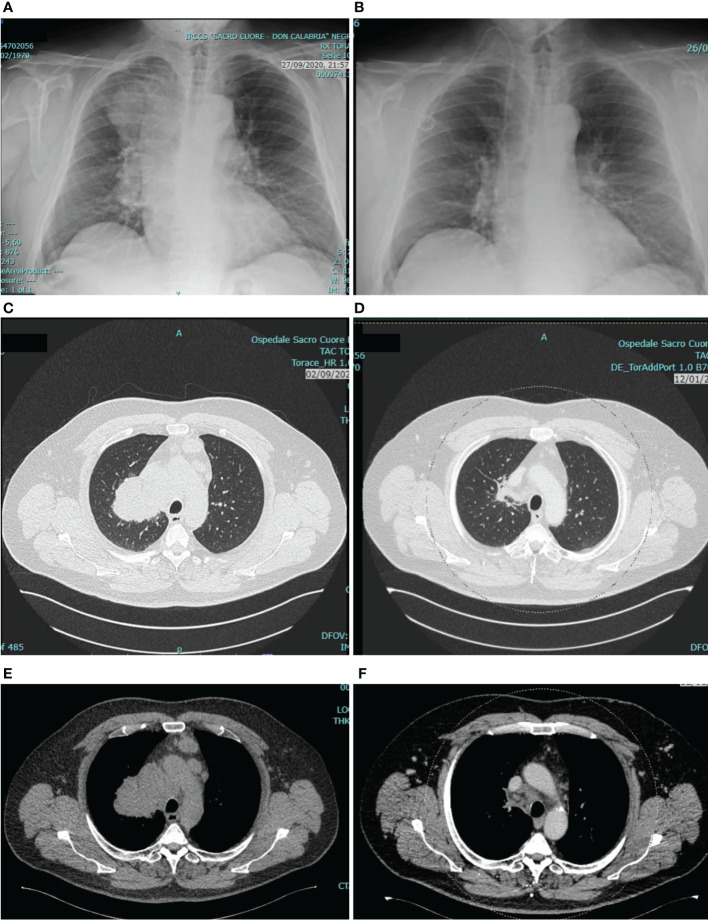
Tumor assessment at baseline and after 4 cycles of pembrolizumab/carboplatin/pemetrexed. The figure shows chest X-ray before **(A)** and after **(B)** treatment, and chest CT scan before **(C, E)** and after **(D, F)** treatment. As compared with baseline, tumor assessment after 4 cycles revealed a deep response of the primary tumor in the upper right pulmonary lobe, and a reduction of para-tracheal and pre-vascular mediastinal pathologic lymph nodes.

From 14th October 2020, the patient underwent first-line treatment for NSCLC with pembrolizumab (200 mg iv), carboplatin (AUC 5 mg/ml/min iv) and pemetrexed (500 mg/m2 iv) every three weeks for 4 cycles. After the first two cycles the patient achieved a dramatic clinical response with complete resolution of the cough and a meaningful improvement of dyspnea and fatigue. Tumor assessment with CT scan after 4 cycles of treatment confirmed an objective partial response with an impressive tumor shrinkage (**Figure 1**). After that, the patient was started on maintenance therapy with pembrolizumab and pemetrexed. until May, 2022 when pemetrexed was discontinued (after 20 maintenance courses) due to G2 protracted fatigue, not associated with significant chemotherapy-induced hematologic toxicity. Although fatigue may occur on a multifactorial basis, including as a side effect of ART as well as of immunotherapy, it was considered mainly related to prolonged pemetrexed exposure. In fact, fatigue resolved after pemetrexed discontinuation. Pembrolizumab alone was continued as maintenance therapy, with further 10 courses administered from May to December 2022, and it is still ongoing, with a ongoing progression-free survival of 27+ months so far. During cancer treatment the patient had a left breast abscess that was successfully managed with oral anti-inflammatory and antibiotic therapy and surgical drainage, and he has experienced no treatment-related adverse events (TRAEs) but the G2 fatigue leading to pemetrexed discontinuation. Particularly, there has been no HIV viral flare or significant worsening of CD4 cell count. In fact, before starting chemo-immunotherapy HIV viral load was undetectable and CD4 cell count was 531/μl (23%) with CD4/CD8 ratio of 0.67, whereas last values while on chemo-immunotherapy treatment were as it follows: viral load undetectable, CD4 cell count 474/μl (24%), CD4/CD8 ratio 0.71.

## Discussion

There is some retrospective evidence on safety and activity of ICIs in PLWHI with NSCLC. A systematic review including 73 cases of cancer PLWHI treated with ICIs (5), 23 of them with NSCLC, reported grade ≥ 3 immune-related adverse events (irAEs) in only 6 out of 70 patients (8.6%). Among 25 patients with paired pre-treatment and post-treatment CD4+ cell counts, the cell counts increased. Objective response rate (RR) among patients with NSCLC was 30%. Similarly, data retrospectively collected from the French CANCERHIV database on 23 PLWHI treated with ICIs for lung cancer (n=21), melanoma (n=1) or head and neck cancer (n=1) reported only two grade 3 irAEs (6). All patients were on antiretroviral therapy (ART) when started ICIs. HIV viral load was undetectable for all the 13 patients with available data about HIV viremia, and only one patient experienced a positive HIV viral load but this occurred after antiretroviral therapy (ART) interruption. CD4+ cell counts had no major changes during treatment with ICIs. In this study, RR among NSCLC was 18%.

More recently, prospective phase 1 and 2 clinical trials also reported data on safety and activity of ICIs in PLWHI with NSCLC. A phase 1 study assessed safety of pembrolizumab as single agent among 30 patients with advanced cancer and HIV infection receiving ART, with a viral load of < 200 copies/mL, and a CD4+ cell count > 100/μl (7). Toxicity profile was acceptable with grade 3 irAEs reported in 6 (20%) patients, no meaningful variations in viral load or need for change of ART. In this study, only one patient with NSCLC was enrolled, obtaining a sustained complete response. A phase 2 study with durvalumab for the treatment of solid tumors in PLWHI enrolled 20 patients, 14 of them with NSCLC (8). According to inclusion criteria, all the patients had to be on ART with undetectable viral load, regardless of their CD4+ T-cell count. No grade ≥ 3 irAEs were observed. CD4+ and CD8+ T-cell remained stable throughout the study, with no significant increase in viral load. Four of 16 response-evaluable patients (25%) had a partial response, and all the responders were NSCLC patients. Interestingly, no correlation of clinical benefit with basal CD4+ or CD8+ T-cell counts was found. Another phase 2 study evaluated nivolumab in 16 PLWHI with NSCLC, with viral load of <200 copies/mL, and no restriction in terms of CD4+ T-cell count (9). Two patients achieved partial response (12.5%), with a disease control rate of 62.5%. Adverse events were generally mild-moderate, with only one case of severe skin toxicity. HIV viral load was stable during treatment, whereas an increase in proliferating CD8+ and CD4+ T-cells was observed after 3 nivolumab cycles in a subgroup of 9 patients.

Taken together, data from retrospective studies and prospective phase 1 and 2 clinical trials reported meaningful activity with an acceptable toxicity profile and no significant risk of viral flare among patients with NSCLC and virologically controlled HIV infection treated with single-agent ICIs. However, data about activity and safety of ICIs plus chemotherapy combinations in this special population are still lacking.

We described a patient with metastatic NSCLC and concurrent HIV infection with undetectable viral load, treated with pembrolizumab, carboplatin and pemetrexed, that achieved a meaningful and durable response, without clinically meaningful TRAEs or HIV flares. This report suggests that chemo-immunotherapy may be active and safe for selected HIV-positive patients with metastatic NSCLC receiving ART with undetectable viral load. Thus, PLWHI with NSCLC should be not excluded from chemo-immunotherapy based only on their viral infection. Data from prospective clinical trials and/or observational registries could contribute to further evaluate chemo-immunotherapy in this special population. At this regard, new clinical trials with ICIs in NSCLC allow the enrollment of patients with HIV infection. For example, phase 3 randomized clinical trials with cemiplimab alone or in combination with chemotherapy for advanced NSCLC did include patients with controlled HIV infection, but results in the HIV-positive population have not been reported yet (10, 11).

## Data availability statement

The raw data supporting the conclusions of this article will be made available by the authors, without undue reservation.

## Ethics statement

Written informed consent was obtained from the individual(s) for the publication of any potentially identifiable images or data included in this article.

## Author contributions

AI wrote the manuscript. All authors contributed to the article and approved the submitted version.
